# Circulating macrophages as the mechanistic link between mosaic loss of Y-chromosome and cardiac disease

**DOI:** 10.1186/s13578-023-01075-7

**Published:** 2023-07-24

**Authors:** Xuehong Xu, Rong Zhou, Qinchun Duan, Yuanlin Miao, Tingting Zhang, Mofei Wang, Odell D. Jones, MengMeng Xu

**Affiliations:** 1 Laboratory of Cell Biology, Genetics and Developmental Biology, Shaanxi Normal University College of Life Sciences and University Hospital Medical Center, 620 West Chang’an, Chang’an District, Xi’an, 710119 China; 2 University of Pennsylvania School of Medicine ULAR, Philadelphia, PA 19144 USA; 3 Department of Pediatrics, Morgan Stanley Children’s Hospital, Columbia University, 3959 Broadway, New York, NY 10032 USA

**Keywords:** Mosaic loss of Y-chromosome (mLOY), Cardiac fibrosis, Hematopoietic macrophage, Profibrotic regulons, Proliferation of cardiac fibroblast, Age-related cardiac disease

## Abstract

**Background:**

Genetics evidences have long linked mosaic loss of Y-chromosome (mLOY) in peripheral leukocytes with a wide range of male age-associated diseases. However, a lack of cellular and molecular mechanistic explanations for this link has limited further investigation into the relationship between mLOY and male age-related disease. Excitingly, Sano et al. have provided the first piece of evidence directly linking mLOY to cardiac fibrosis through mLOY enriched profibrotic transforming growth factor β1 (TGF-β1) regulons in hematopoietic macrophages along with suppressed interleukin-1β (IL-1β) proinflammatory regulons. The results of this novel finding can be extrapolated to other disease related to mLOY, such as cancer, cardiac disease, and age-related macular degeneration.

**Results:**

Sano et al*.* used a CRISPR-Cas9 gRNAs gene editing induced Y-chromosome ablation mouse model to assess results of a UK biobank prospective analysis implicating the Y-chromosome in male age-related disease. Using this in vivo model, Sano et al*.* showed that hematopoietic mLOY accelerated cardiac fibrosis and heart failure in male mice through profibrotic pathways. This process was linked to monocyte-macrophage differentiation during hematopoietic development. Mice confirmed to have mLOY in leukocytes, by loss of Y-chromosome genes Kdm5d, Uty, Eif2s3y, and Ddx3y, at similar percentages to the human population were shown to have accelerated rates of interstitial and perivascular fibrosis and abnormal echocardiograms. These mice also recovered poorly from the transverse aortic constriction (TAC) model of heart failure and developed left ventricular dysfunction at higher rates. This was attributed to aberrant proliferation of cardiac MEF-SK4 + fibroblasts promoted by mLOY macrophages enriched in profibrotic regulons and lacking in proinflammatory regulons. These pro-fibrotic macrophages localized to heart and eventually resulted in cardiac fibrosis via enhanced TGF-β1 and suppressed IL-1β signaling. Furthermore, treatment of mLOY mice with TGFβ1 neutralizing antibody was able to improve their cardiac function. This study by Sano et al*.* was able to provide a causative link between the known association between mLOY and male cardiac disease morbidity and mortality for the first time, and thereby provide a new target for improving human health.

**Conclusions:**

Using a CRISPR-Cas9 induced Y-chromosome ablation mouse model, Sano et al. has proven mosaic loss of Y-chromosome in peripheral myeloid cells to have a causative effect on male mobility and mortality due to male age-related cardiac disease. They traced the mechanism of this effect to hyper-expression of the profibrotic TGF-β1 and reduced pro-inflammatory IL-1β signaling, attenuation of which could provide another potential strategy in improving outcomes against age-related diseases in men.

A correlation between peripheral leukocyte mosaic loss of Y-chromosome (mLOY) and many life-threatening diseases *i.e.* age-related diseases and oncologic diseases including colorectal, bladder, lung, and androgen associated cancers such as prostate and testicular germ cell tumors in men has been revealed for over a decade [1–7]. These diseases cover a wide range of organs, from age-related macular degeneration to multiple oncogenic tissues [1–7]. MLOY had also been implicated in hypertension and cardiovascular disease, the leading cause of death in men [8, 9]. Cardiac fibrosis alone causes more than 800,000 deaths annually and plays a key role in all cardiovascular disease (CVD), which is responsible for over 17.5 million annual deaths worldwide [10]. Although significant resources have been directed at CVD over the past four decades, there is still a lack of effective treatments targeting the fibrotic process and a lack of understanding behind the mechanism of cardiac fibrosis. Here we highlight a recent study identifying a mechanism behind accelerated cardiac fibrosis in men with leukocyte mLOY.

Using a mouse model of Y-chromosome deletion to confirm UK Biobank prospective study findings of increased all-cause and cardiac morbidity and mortality in men with a higher proportion of mLOY (UK Biobank resource/application no. 18,623, 21,552 and 61,272) [11, 12], an international collaborative revealed the causal and mechanistic relationships behind mLOY and cardiac fibrosis and mortality [11, 13]. Using CRISPR-Cas9 to target repeat sequences around the Y chromosome centromere using the Y chromosome specific guide RNAs (gRNAs- empty control, LOY-gRNA1- Y specific guide one and LOY-gRNA2-Y specific guide two) and tomato red fluorescent protein (tRFP) marker, Sano et al*.* generated hematopoietic Y chromosome ablation mice generated by lentivirus transduction. Bone marrow cells successfully transduced by the lentivirus were selected by their tRFP positivity and collected from the reconstituted mice [13, 14]. Using this approach, the efficiency of Y chromosome ablation reached 95% for LOY-gRNA1 and 80% for LOY-gRNA2 as detected by tRFP. This model was able to match the percentage of mLOY white blood cells (as) found in the human population of 49–81% [11, 13]. The validity of their model was confirmed by a lack of expression of Y-chromosome genes *Kdm5d*, *Uty*, *Eif2s3y*, and *Ddx3y* in tRFP + leukocytes. They were then able to show that mLOY mice had accelerated age-related cardiomyopathy and echocardiogram findings consistent with heart failure. These mLOY mice also had poorer outcomes following the transverse aortic constriction (TAC) technique, a commonly used model of non-ischemic heart failure.

Compared to wildtype mice after TAC, TAC mLOY mice had significantly increased heart weight to tibia length ratio, atrial natriuretic peptide expression, and interstitial and perivascular fibrosis, all indicating advanced heart failure. TAC mLOY mice were found to have aberrant proliferation of cardiac MEF-SK4 + fibroblasts. This along with no change in the number or size of cardiomyocytes, indicted that mLOY related heart failure is through increased cardiac fibrosis. These findings were consistent with UK Biobank data indicating an increased risk of death due to cardiovascular disease in men with a high proportional of mLOY leukocytes [11, 13]. Several studies have previously linked LOY to coronary artery disease (CAD). In early 2012, an inheritance cohort study proved Y chromosome function to be strongly linked to CAD [15]. Using 11 genetic markers specific to the Y chromosome, Charchar et al. investigated the correlation between CAD and the Y chromosome in 3233 British men included in the British Heart Foundation Family Heart Study (BHF-FHS), West of Scotland Coronary Prevention Study (WOSCOPS), and Cardiogenics Study. Using the data from these three cohorts, they were able to unveil Y chromosome polymorphisms strongly associated with CAD [15]. Even accounting for conventional CAD risk factors such as BMI, hypertension, and hypercholesterolemia, male-specific region of Y chromosome (MSY haplogroup I) has been associated with ~ 50% increase in CAD risk compared to other Y chromosome lineages [15]. Furthermore, through the Cardiogenics cohort, they identified this relationship to be mediated through dysregulation of immunity and inflammation facilitated by circulating hematogenous macrophages. Their transcriptome analysis isolated 19 pathways with strong differential expression including down-regulated pathway related to haemopoietic cell lineage and up-regulated pathways strongly associated with high risk of heart dysfunctions such as arrhythmogenic right ventriclar cardiomyopathy, dilated cardiomyopathy and hypertrophic cardiomyopathy [15]. These findings were later confirmed by Eales et al., which analyzed over 129,000 men from the UK Biobank and confirmed that MSY haplogroup I had a 11% increased risk of CAD in comparison to all other haplotypes combined. They also found expression of the Y chromosome gene, *UTY*, as the only hematogenous gene expression associated with MSY haplogroup I. Macrophage involvement was confirmed when experimental reduction of *UTY* expression in macrophages to significantly reduce the immune costimulatory signal important in both early and late stage atherosclerosis development [16].

Building off these findings, using gene network analysis on single cell regulatory network inference and clustering (SCENIC), Sano et al*.* were able to provide exact mechanistic evidence to how mLOY in leukocytes were able to affect cardiac function [11, 13]. During development of the hematopoietic macrophages-monocyte lineage, they found mLOY leukocytes to be simultaneously enriched in profibrotic regulons of TGF-β1 signaling along with suppressed IL-1β proinflammatory regulons. This results in circulating pro-fibrotic macrophages which localized to the heart during stress (natural aging process) and injury (TAC) and predisposing organ repair down the pro-fibrotic pathway.

These findings by Sano et al*.* can be added to by Hulsmans et al*.* who showed hematopoietic macrophage can localized to the heart and naturalize in the atrial-ventricle (AV) node by binding to conductive cardiomyocytes. These resident macrophages are critically involved in cardiac function and regulate electrophysiological activity of the heart via the gap-junction protein connexin 43 (Cx43) [18]. Following the natural connection between these two studies, it is reasonable to postulate that hematopoietic mLOY may also produce mLOY macrophages that are contributing to arrhythmias commonly found in patients with cardiac failure. This would suggest two separate pathways through which hematopoietic mLOY could lead to cardiac disease in men (Fig. 1): (1) mLOY leukocytes leads to pro-fibrotic regulons that promote cardiac fibrosis during times of stress and injury leading to cardiac dysfunction, and (2) mLOY macrophages localized to the AV node and disturb electroconductive gap-junctions, and leading to arrhythmia along with cardiac fibrosis syndrome.

**Fig. 1:**
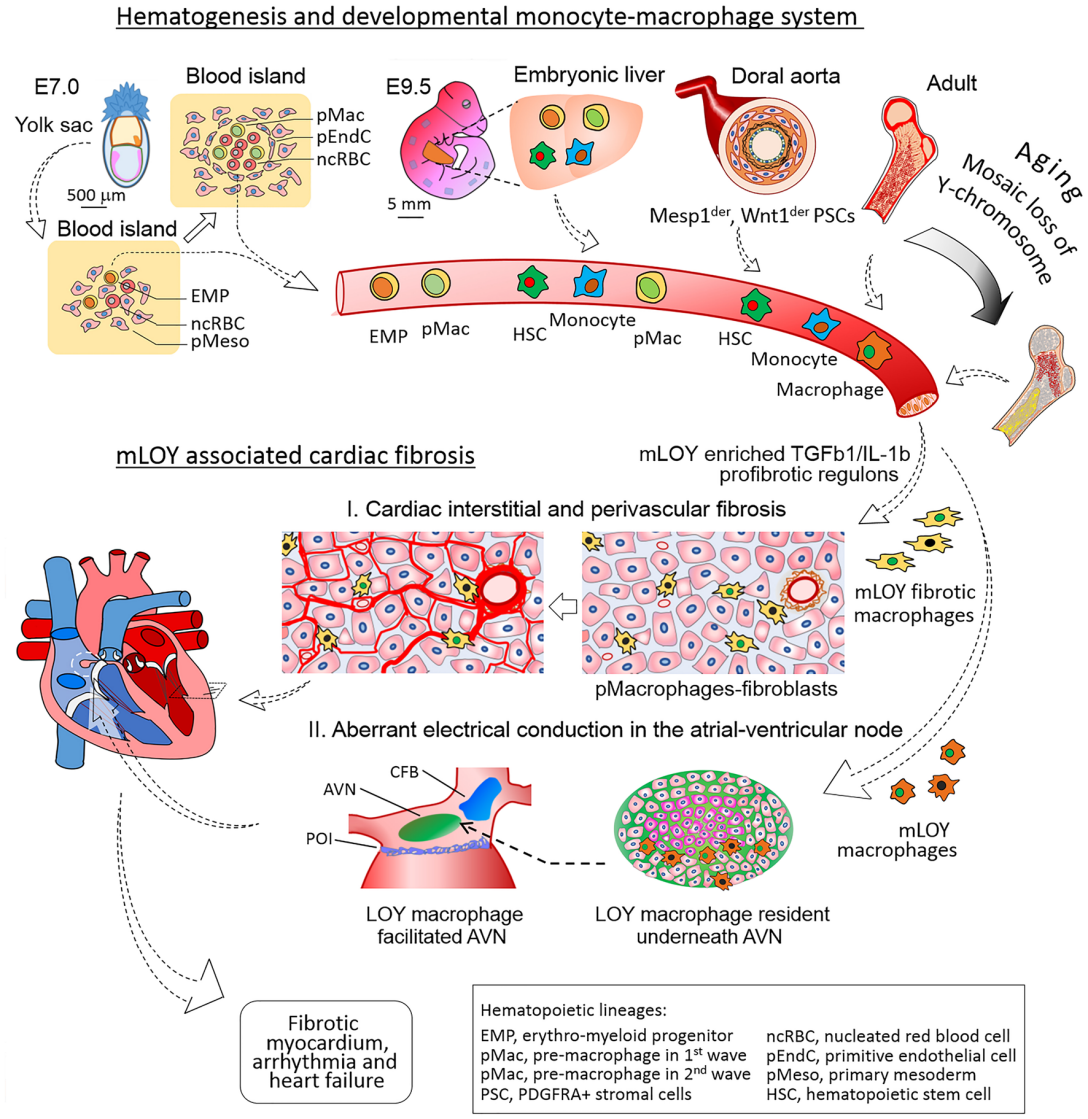
Mechanism behind the association of mLOY with cardiac fibrosis through cardiac localization of hematogenic mLOY macrophages. Although hematopoietic stem cells originally begin in the embryonic yolk sac, then the liver and dorsal aorta, by adult life, hematopoiesis lies in the bone marrow. Thus, mosaic loss of Y-chromosome in peripheral leukocytes originate in marrow myeoloid progenetors. The resultant mLOY leukocytes are then distributed throughout the body, including in to the myocardium and atrial-ventricular node, where their enrichment of TGFb1 expression and suppression of IL-1b expression promotes pro-fibrotic response to stress resulting in heart failure and arrhythmia. AVN, Atrial-ventricle node; CFB, Central fibrous body; POI, Plane of insulation

The adult mammal heart possesses three distinct macrophage populations (or three waves) which originate from different embryonic progenitors, including primitive yolk sac macrophages on embryonic day 7.0, fetal liver monocyte progenitors on embryonic day 9.5, and the traditionally associated hematogenous monocyte-macrophage system (Fig. 1) [19–22]. In the setting of cardiac ischemia/reperfusion injuries, bone marrow monocyte production increases to produce circulating monocyte populations, which in turn localize to a myocardia macrophage niche in which these monocytes support myocardial recovery. However, the macrophages localized during embryogenesis also play a role myocardial recover. Any LOY during initial embryogenic differentiation of these cells would naturally affect the function of all macrophages in this lineage. Thus LOY loss in either embryogenic or mature hematopoietic macrophage populations would logically lead to suboptimal macrophage function and poor cardiac recovery in the adult patient [23–25]. A recent study using long term in vivo tracing of yolk sac embryonic hematopoietic stem cells (HSCs) traced these cells to the aorta-gonad-mesonephros (AGM), umbilical vessels, and other extraembryonic tissues. Importantly, these cells retain some of their hematopoietic nature even in adult mouse [26]. Samokhvalov showed yolk sac cells expressing *Runx1* at embryonic day 7.5 later develop into fetal lymphoid progenitors and adult HSCs [26]. Using in vivo genetic tracing Yokomizo et al*.* recent showed fetal yolk sac derived HSCs expressing the liver specific transcription factor hepatic leukaemia factor (HLF) to be minimally involved in fetal hematooiesis, and instead is key in developing post-gestational intra-arterial hematopoietic clusters [27]. These results have been further confirmed using the zebrafish model [25]. Further implicating the importance of embryonic HSC in adult hematopoiesis, using Calreticulin knock-down and embryonic macrophage depletion approaches, Wattrus et al. demonstrated embryonic macrophage to be essential quality controls for establishing adult hematopoiesis [28].

Although the exact link between mLOY macrophages and cardiac conduction system is currently unknown, findings by Hulsmans et al*.* have identified a possible link. They identified a macrophage niche underneath atrial-ventricle (AV) node that puts localized macrophage directly in contact with the electroconductive cells in cardiac conduction system [18]. They found elongated cardiac macrophages expressing connexin43 to be densely intersperse with conducting cell in the distal atrioventricular node. Cardiac macrophage experimentally coupled to spontaneously beating cardiomyocytes via connexin-43-containing gap junctions were found to depolarize in a synchronized fashion with cardiomyocytes. Importantly, changing the membrane potential of these macrophages affected the resting potential of the associated cardiomyocytes and affected their repolarization, and thus their conduction ability in situ. Furthermore, conditional deletion of connexin43 in macrophages and congenital lack of macrophages delayed atrioventricular conduction in mice, resulting in these mice developing progressive atrioventricular block. These findings implicate that macrophage abnormality alone without intrinsic cardiac abnormality can induce aberrant cardiac conductions.

The heart is normally made of multiple heterogeneous population of cells predominantly consisting of cardiomyocytes, endothelial cells, fibroblasts, pericytes, and smooth muscle cells. Although the immune system is not classically associated with the heart, many immune cells are also found a vital component of the healthy heart [29–31]. What all these cell types, including macrophage, have in common is their response to TGF-β signaling. TGF-β signaling affects all cell types during the cardiac injury, repair and remodeling process involved in CVD [32]. TGF-β signaling is Smad-dependent. The tetrameric TGF-β receptor complex is composed of two copies each of the type-I and type-II receptors. Phosphorylation on specific sites of the type-I receptors activates their kinase domains, resulting in phosphorylation of R-Smads (regulated-SMADs) such as Smad2 and Smad3, and triggering the formation of a trimeric Smad complex with R-Smads and co-Smad (common-Smads) such as Smad4. This cytosolic phosphorylated Smad complex then transverses nuclear pore-baskets to interact with various transcription regulators that further control the transcription of specific target genes involved in cardiac fibrosis and ischemic remodeling [33, 34] (Fig. 2).

**Fig. 2:**
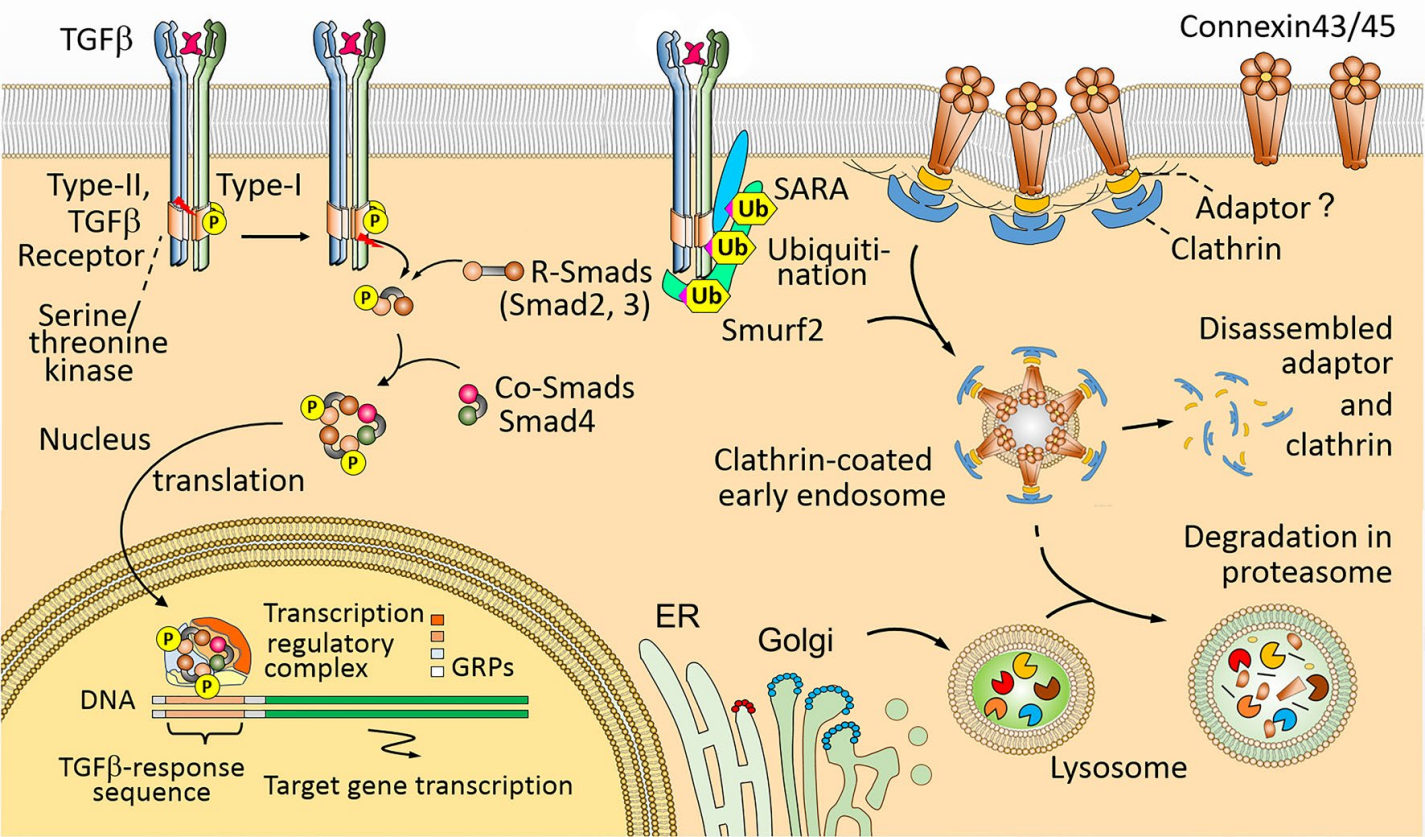
Hypothesized mechanism of *SARA*-Smurf2 associated Connexin43/45 endocytosis and degradation via aberration of TGF-β signaling may critically cause the mosaic loss of electroconductive gap-junction protein Connexin43/45 in aging men with mLOY. An anchoring protein *SARA* (*Smad anchor for receptor activation*) binds to TGF-β receptors and activate ubiquitylation via Smurf2 (Smad ubiquitination regulatory factor-2). The Clathrin-coated early Connexin43/45 endosome then combines with cytoplasmic lysosome generated by ER-Golgi complex to form the late endosome, in which the gap-junction protein Connexin43/45 undergo degradation. This results in disassembly of gap-junctions, which could result in impaired cardiac conduction and subsequent cardiac arrhythmia. ER, endoreticulum; GRPs, Gene regulatory proteins; R-Smads, receptor-regulated Smads; C-Smads, common-partner Smads

In the heart, mLOY mediated cardiac disturbance of the electroconductive gap-junction protein could occur through TGF-β signaling. Multiple studies in other cell types, such as osteocytes, have shown TGF-β1 mediated effects on cell–cell communication via gap-junction proteins such as connexin43 and N-cadherin [35–38]. In cultured osteocytes, Fykerud et al. demonstrated Smad ubiquitination regulatory factor-2 (Smurf2) to affect gap-junctional intercellular communication via endocytosis and degradation of connexin43 and connexin45 [39].

Although no publication currently reports how Smad-TGF-β1 signaling regulates electroconductive gap-junction proteins in the heart, since macrophages have been shown to communicate with the AV node cardiomyocytes through connexin43, and connexin43 is known to be regulated by TGF-β signaling in other cell types, regulation of mLOY in macrophages through TGF-β signaling is possible. If this hold true, abnormal Smad-TGFβ signaling can affect the cardiomyocyte in two ways: (1) mLOY macrophages preferentially activate the R-Smads complex to form transcription regulatory complexes that known to trigger the pro-fibrotic axis in cardiomyocytes, leading to preferential cardiac fibrosis of surrounding cardiomyocytes; and (2) mLOY in AV node-integrated macrophages cause pathological Smad-TGF-β1 signaling, which activates the Smurf2 pathway in surrounding cardiomyocytes, leading to endocytosis and degradation of gap-junction proteins like connexin43, eventually leading to loss of vital gap junction signaling, resulting in interrupted conduction and cardiac arrhythmia (Fig. 2).

Although peripheral mosaic loss of Y-chromosome in leukocytes/myeloid cells have been correlated with many age-related diseases in men [1, 4, 5, 9], this is the first time an animal model of peripheral mLOY has definitively showed a causative relationship [11, 12]. Through their CRISPR-cas9 hemopoietic Y-chromosome deletion mouse model of mLOY, Sano et al*.* have shown the mechanism behind mLOY and cardiac disease to be increased cardiac fibrosis due to pro-fibrotic leukocytes generated by mLOY. This mechanistic understanding not only provides a potential therapeutic strategy against the top cause of mortality in men, but also provides a possible treatment against other disease related to mLOY, such as cancer and age related macular degeneration.

LOY is not limited to just macrophages in ageing humans. Forsberg et al. has shown a relationship between male carcinogenesis and progressive accumulation of LOY in all cell types with increasing age [7]. Likewise Thompson et al. have suggested that macrophage LOY is simply a biomarker of general genetic instability and goes in hand with mLOY throughout the body [18]. Logically, these concomitant mutations in the affected organ result in subsequent functional consequences and contribute to age-related illness like cancer and cardiac disease. However, the recovery of these already cells after an external insult such as ischemia or reperfusion likely requires the assistance of the immune system for clearance of dead cells and recovery of injured cells. Thus, macrophages LOY leading to reduced or aberrant function likely impair immune mediated repair of injured tissues. This theory is supported by Hulsmans et al*.* finding that genetically modified macrophages can localized to the heart and naturalize in the atrial-ventricle (AV) node to regulate electrophysiological activity [17]. This is further supported by Sano et al. findings clearing showing macrophage LOY negatively impacts healing [11].

## Data Availability

Not applicable.

## Abbreviations

AV: Atrial-ventricle
AVN: Atrial-ventricle node
AGM: Aorta-gonad-mesonephros
BHF-FHS: British Heart Foundation Family Heart Study
CAD: Coronary artery disease
CVD: Cardiovascular disease
CFB: Central fibrous body
C-Smads: Common-partner Smads
EMP: Erythro-myeloid progenitor
ER: Endoreticulum
IL-1β: Interleukin-1β
GRPs: Gene regulatory proteins
HLF: Hepatic leukaemia factor
HSC: Hematopoietic stem cell
mLOY: Mosaic loss of Y-chromosome
ncRBC: Nucleated red blood cell
MSY: Male-specific region of Y chromosome
pEndC: Primitive endothelial cell
POI: Plane of insulation
pMac: Pre-macrophage
pMeso: Primary mesoderm
PSC: PDGFRA + stromal cells
R-Smads: Receptor-regulated Smads
SARA: Smad anchor for receptor activation
Smurf2: Ubiquitination regulatory factor-2
SCENIC: Single cell regulatory network inference and clustering
TAC: Transverse aortic constriction
TGFβ1: Transforming growth factor β1
WOSCOPS: West of Scotland Coronary Prevention Study
